# Infrapatellar Fat Pad Stem Cells: From Developmental Biology to Cell Therapy

**DOI:** 10.1155/2017/6843727

**Published:** 2017-09-06

**Authors:** Ronaldo J. F. C. do Amaral, Henrique V. Almeida, Daniel J. Kelly, Fergal J. O'Brien, Cathal J. Kearney

**Affiliations:** ^1^Tissue Engineering Research Group, Department of Anatomy, Royal College of Surgeons in Ireland, Dublin, Ireland; ^2^CNC, Center for Neuroscience and Cell Biology, University of Coimbra, 3004-517 Coimbra, Portugal; ^3^Trinity Centre for Bioengineering, Trinity Biomedical Sciences Institute, Trinity College Dublin, Dublin, Ireland; ^4^Department of Mechanical and Manufacturing Engineering School of Engineering, Trinity College Dublin, Dublin, Ireland; ^5^Advanced Materials and Bioengineering Research Centre (AMBER), Trinity College Dublin & Royal College of Surgeons in Ireland, Dublin, Ireland

## Abstract

The ideal cell type to be used for cartilage therapy should possess a proven chondrogenic capacity, not cause donor-site morbidity, and should be readily expandable in culture without losing their phenotype. There are several cell sources being investigated to promote cartilage regeneration: mature articular chondrocytes, chondrocyte progenitors, and various stem cells. Most recently, stem cells isolated from joint tissue, such as chondrogenic stem/progenitors from cartilage itself, synovial fluid, synovial membrane, and infrapatellar fat pad (IFP) have gained great attention due to their increased chondrogenic capacity over the bone marrow and subcutaneous adipose-derived stem cells. In this review, we first describe the IFP anatomy and compare and contrast it with other adipose tissues, with a particular focus on the embryological and developmental aspects of the tissue. We then discuss the recent advances in IFP stem cells for regenerative medicine. We compare their properties with other stem cell types and discuss an ontogeny relationship with other joint cells and their role on *in vivo* cartilage repair. We conclude with a perspective for future clinical trials using IFP stem cells.

## 1. Introduction

Cell-based approaches are increasingly gaining attention in the development of treatments for articular cartilage defects [1–4], especially since the clinical application of autologous chondrocytes for articular cartilage repair in 1994 (autologous chondrocyte implantation, ACI) [5, 6]. However, the development of a regenerated cartilage that fully recapitulates the native tissue still eludes us. It is therefore unsurprising that a full consensus has not yet been reached on the optimum cell source for cartilage tissue regeneration [7, 8].

Some of the most frequently studied cells include mature chondrocytes, chondrocyte progenitors, embryonic stem cells (ESC), induced pluripotent stem cells (iPS), and mesenchymal stem cells (MSC). Mature chondrocytes, such as those currently used in ACI, have led to improved clinical outcomes [5], although there are challenges associated with their isolation, culture, donor-site morbidity, and dedifferentiation [9–11]. Tissue-specific progenitor cells found in the perichondrium [12, 13], periosteum [14], and in normal or osteoarthritic (OA) cartilage itself [15–17] are being actively explored as substitutes to mature chondrocytes. Studies on the chondrogenic differentiation of ESC and iPS have shown these cell types are emerging as potential future cell sources for cartilage repair [18]; however, ethical and/or safety issues remain (e.g., tumor formation) [19]. Given their availability and chondrogenic potential, MSC—primarily from the bone marrow but also from adipose tissue—have emerged as the most promising cell source to regenerate articular cartilage [20–22].

Interestingly, MSC isolated from tissues within the articular joint possess superior chondrogenic capacity when compared to the bone marrow or subcutaneous adipose tissue-derived MSC [23]. Specifically, MSC can be isolated from the synovial fluid [24, 25], synovial membrane [26, 27], and the infrapatellar fat pad (IFP) [28–32]. MSC isolated from the synovial fluid or the synovial membrane have been previously discussed in another review paper [33], and the latter have already been investigated in a clinical study, where significant improvements in clinical outcomes were demonstrated including improved MRI scores (from 1.0 ± 0.3 to 5.0 ± 0.7, median ± 95% CI) which grade for “degree of defect repair and filling of the defect” [34], Lysholm knee scores (from 76 ± 7 to 95 ± 3, median ± 95% CI) which grade “patients' own opinion of function” [35] and histological qualitative assessments [27]. Although very few clinical trials have been reported so far employing IFP stem cells [36, 37], this review will outline how these cells could be a very promising source for cartilage regeneration. First, we will discuss IFP as a tissue source, anatomically and developmentally. Next, we will describe the latest advances in analyzing the therapeutic potential of IFP stem cells for cartilage regeneration. Finally, we will compare IFP stem cells to other cell types in the joint, suggesting their main role in the maintenance of joint homeostasis. In the conclusions and future perspectives section, we will motivate the use of IFP cells in future clinical trials.

## 2. The IFP Structure and Development

In order to put forward the IFP as a promising cell source for cartilage regeneration, it is important to understand its anatomical characteristics, as well as its developmental origin. As an adipose tissue within the joint, the IFP can be easily harvested arthroscopically or during open knee surgery [38]. The IFP is an intracapsular structure in the anterior knee compartment, composed of approximately 20 cm^3^ of adipose tissue, or slightly larger in patellofemoral OA joints [39–41]. As it is lined on its deep surface by the synovial membrane, it is classified as an extrasynovial structure. The IFP lies inferior to the patella and posteriorly extends into the infrapatellar plica (IPP) (ligamentum mucosum), which inserts into the anterior border of the intercondylar notch [42]. The infrapatellar plica is, together with the suprapatellar and mediopatellar, one of the three plicas in the knee. These plicas are believed to be synovial fold remnants from the incomplete resorption of the synovial septa during the embryological development of the knee [43].

Although generally considered to participate in the biomechanics of the knee [44], the exact roles of IFP in articular physiology have not been fully elucidated [45]. In 1691, it was originally proposed by Havers et al. that synovial fat pads were responsible for the secretion of synovial fluid. However, it is now believed that they simply occupy space in the joint, maintaining the articular cavity, allowing the synovial fluid to circulate over the joint, and contributing to lubrication [46], with the contribution to lubrication attributed to increased synovial surface resulting from their anatomical location [47]. Participation of the IFP in shock absorption has also been proposed [48]. Interestingly, the weight of the synovial fat pads are unrelated to the state of nutrition, unless in extreme emaciation [49]. Even under starvation conditions, with the elimination of the subcutaneous adipose tissue, the IFP may be preserved [50]—this biological drive underscores its importance in the knee joint. As discussed later in this review, we believe this biological drive results from IFP stem cells' role in tissue maintenance and repair.

**Figure 1:**
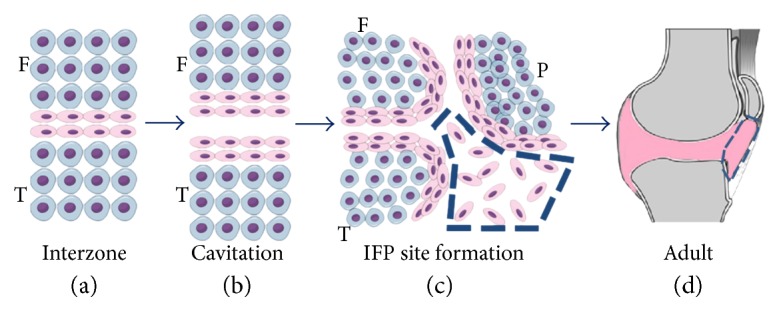
Ontogeny of the infrapatellar fat pad (IFP). During embryonic development, (a) a dense mesenchyme tissue arises between the chondrification of the femur (F) and tibia (T), the interzone (flattened cells in pink). (b) This is followed by a cavitation in between this region. (c) By the 9th week of human development, a triangular space composed of a mesenchymal tissue becomes visible below the patella (P) (highlighted by the blue dashed). (d) In adulthood, interzone cells will have contributed to several joint structures (pink), including the IFP (highlighted by the blue dashed). This image was made using https://MindTheGraph.com.

Besides its anatomical position, the embryonic origin of the IFP also highlights its potential. Synovial joints develop through the formation of an interzonal layer of flattened cells within a mesenchymal condensation, which is responsible for the cavitation and formation of the joint tissues [51]. Specifically, during human knee formation, at the 9th week of development—when the chondrification of the patella, femur, and tibia has already begun but prior to the menisci maturation and ossification—the chondral anlagen—a triangular space occupied by a mesenchymal tissue—appears below the patella. It is thought that this is the site of formation of the future IFP [52]. The cells from the interzone will further contribute to the development of the epiphyseal articular chondrocytes, ligaments, menisci, synovial lining, and fat pad [53–57] (Figure 1).

## 3. The IFP as an Adipose Tissue

Although the exact roles of the IFP and its development are not yet fully understood, it is important to highlight its nature as an adipose tissue and, more specifically, as an elastic adipose tissue, due to its orcein-stained elastic-fiber content [49]. Traditionally, the adipose tissue has been identified as a metabolic tissue responsible for storing energy in the form of fat. However, more recently, due to the description of adipokines and their regulation of appetite and participation in inflammation and vascular diseases, the adipose tissue is now also regarded as an endocrine tissue [58, 59]. Indeed, the adipose tissue can be divided into brown (BAT) and white (WAT) types, which are further divided into subcutaneous and visceral adipose tissue. WAT is commonly associated with energy storage, while BAT with energy dissipation in the form of heat [60]. In a similar way, subcutaneous adipose tissue is more predisposed to storing free fatty acids and triglycerides, while visceral adipose tissue is more cellularized, vascularized, innervated, and therefore more metabolically active and predisposed to insulin resistance [61]. Different sites of the adipose tissue therefore present different physiological properties [62].

Although all adipose tissues possess a mesodermal origin, different stem cell populations give rise to visceral and subcutaneous adipose tissue [63, 64], for example mesothelial cells originated mainly from the lateral plate mesoderm strongly contribute to the formation of visceral adipocytes, while paraxial mesoderm and neural crest contribute to the formation of mesenchymal/mesodermal stem cells that originate subcutaneous adipocytes [65, 66]. Based on this delineation, it is important to note that the IFP should be evaluated as an adipose tissue with singular characteristics. It does not correlate with visceral adipose tissue since its origin is not related in any way to the formation of visceral structures and has never presented a mesothelium cover. On the other hand, it is not functionally similar to subcutaneous adipose tissue either; for instance, IFP from obese patients secretes different levels of inflammatory molecules and adipokines (e.g., higher levels of IL-6, soluble IL-6 receptor and adiponectin, and lower levels of leptin), and expresses lower levels of lipid metabolism-related genes compared to subcutaneous adipose tissue [67].

## 4. Adipose-Derived Stem Cells

Mesenchymal stem-like cells with multilineage differentiation capacities were first isolated from the human subcutaneous adipose tissue obtained after the enzymatic digestion of lipoaspirate samples in 2001 [68]. This was in accordance with later findings that MSC with tissue/organ-specific characteristics could be found in virtually all organs, occupying a perivascular niche [69, 70]. Further investigation confirmed that although MSC derived from both subcutaneous adipose tissue and the bone marrow are multipotent, the bone marrow-derived MSC are more committed to osteogenic and chondrogenic lineages, while adipose-derived stem cells are more committed to the adipogenic lineage [71]. Moreover, while CD34 is not expressed by the bone marrow-derived MSC, it is only the CD34+ cells in the adipose tissue which are capable of multilineage differentiation and of forming fibroblastic colony-forming units (CFU-F) [72]. More recently, it has been proposed that four different nonhematopoietic (CD45−) progenitor populations exist in adipose tissue: endothelial progenitors (CD146+/CD31+/CD34+); pericytes (CD146+/CD31−/CD34−), which are more naïve; a transit amplifying progenitor population (CD146+/CD31−/CD34+); and a more adipogenic-committed supra-adventitial adipose stromal cell population (CD146−/CD31−/CD34+) [73].

In 2013, the International Federation for Adipose Therapeutics and Science (IFATS) and the International Society for Cellular Therapy (ISCT) published a joint statement on some definitions regarding adipose-derived stem cells. For instance, marker expression profiles of cells from the stromal vascular fraction (SVF) and the adipose tissue-derived stromal cells (ASC) have been defined. The SVF comprises the cell populations obtained after enzymatic digestion of the adipose tissue, separated from the mature adipocytes through centrifugation, such as endothelial cells, erythrocytes, fibroblasts, lymphocytes, monocytes/macrophages, pericytes, and stem/progenitor populations; while the ASC comprises the adherent cells populations obtained from the SVF [74].

## 5. The IFP Stem Cells

In 1996—before the first isolation of adipose-derived stem cells (2001)—Maekawa et al. described a population of fibroblasts that are a “kind-of a stem cell” in the synovial tissue near the IFP. The cells described reside in a perivascular niche, expressing fibronectin and laminin, and are associated with small vessels. They participate in anterior cruciate ligament (ACL) repair after injury, by secreting extracellular matrix (ECM) components such as collagen type I and III. Moreover, these cells can also differentiate into surface-lining phagocytic fibroblasts [75]. To our knowledge, this was the first report of a stem cell-like population related to the IFP.

More recently, multipotent stem cells from IFP have been isolated and characterized as CD9+, CD10+, CD13+, CD29+, CD44+, CD49e+, CD59+, CD105+, CD106+, and CD166+ with the ability to differentiate into chondrogenic, osteogenic, and adipogenic lineages under the appropriate stimulation *in vitro*. Under chondrogenic stimulation, the cells did not produce collagen type X, a marker of hypertrophy [76]. Since then, many studies have confirmed IFP stem cells' chondrogenic capacity in different *in vitro* and *in vivo* models [28–30, 32, 51, 77–81].

Continuing the characterization studies, Hindle et al. distinguished two populations within the IFP stem cells: the pericytes (CD31−/CD34−/CD45−/CD146+) and adventitial cells (CD31−/CD34+/CD45−/CD146−), corresponding to 3.8% and 21.2% of the isolated cells at the stromal vascular fraction (SVF). The two mixed populations were termed “perivascular stem cells” (PSC). The total adherent population was termed “MSC.” Interestingly, both PSC and MSC derived from IFP showed superior chondrogenic capacity compared to the bone marrow-derived MSC. Additionally, comparing the two populations from IFP, it was found that PSC were superior to MSC [82]. Additionally, 3G5 (a pericyte marker) has been detected in IFP cells in situ. Situated in the adventitia of small blood vessels, 1–20% of cells retained 3G5 expression both in mixed and clonal population after expansion in culture [51].

When compared with other cell types, IFP stem cells were found to retain their chondrogenic potential for a longer period than chondrocytes obtained from OA articular cartilage [83]. Compared with subcutaneous adipose tissue-derived stem cells, IFP stem cells presented with a similar ability to form CFUs; however, they expressed higher levels of chondrogenic and osteogenic genes [84]. Compared with the bone-marrow MSC, IFP stem cells generated more cartilaginous ECM in pellet cultures and expressed higher levels of chondrogenic genes [82]. In one study, IFP stem cells were compared to the bone marrow-derived MSC and synovium-derived stem cells (SDSC) [85]. Importantly, SDSC had been reported to present enhanced chondrogenic potential compared to the bone marrow, adipose, and muscle MSC both *in vitro* [86] and *in vivo* [23]. Although SDSC generated the most functional and mechanically stable cartilaginous tissue *in vitro*, none of the cell types generated stable cartilage after subcutaneous implantation *in vivo*. Nevertheless, the authors stated that it is not possible to fully conclude that SDSC possess higher chondrogenic capacity compared to the other cell types, since different culture conditions may interfere with each cell's chondrogenic potential [85]. For instance, the application of physiological levels of hydrostatic pressure (HP) further enhances IFP stem cells chondrogenic capacity, as well as maintain their potential after removal of TGF-*β*3 stimuli [87]. Moreover, when exposed to dynamic compression and a gradient oxygen tension [88] or cultured in decellularized cartilage explants [89], IFP stem cells produced cartilaginous ECM with zonal architecture that resembled native articular cartilage.

It is possible that specific subpopulations in the heterogeneous IFP stem cell population may possess an even greater chondrogenic potential. A subpopulation of freshly isolated, that is not expanded *in vitro*, CD44+ cells (approximately 10% of the entire population) showed an impressive capability to synthesize sGAG and collagen type II *in vivo* when seeded on a cartilage ECM-derived scaffold and subcutaneously implanted [28]. The idea of using an enriched population with increased chondrogenic capacity, without the need of culture expansion, is particularly promising for clinical trials. A study comparing donor-matched articular chondrocytes, bone marrow, IFP, and subcutaneous adipose tissue stem cells also suggested that CD49c and CD39 expression positively correlate to an enhanced in vitro chondrogenic potential, besides suggesting IFP stem cells as the best stem cell alternative to chondrocytes, followed by the bone marrow and subcutaneous adipose tissue [90].

Recently, IFP stem cells were also compared to synovial fluid stem cells (SFSC). The chondrogenic capacity of both was considered similar *in vitro*, although the adipogenic and osteogenic potential of IFP stem cells was greater. Moreover, the expression of CD34 was detected in 30.1% ± 18.6% of passage 3-4 IFP stem cells. As mentioned previously, this marker is also present in adipose-derived stem cells and is related to their multilineage potential [72]. Finally, both populations presented CD14 positivity, a marker of macrophage lineage, although there was some variation among donors (30.5% ± 30.3% for IFP stem cells and 7.4% ± 7.2% for SFSC) [91].

A possible strategy in the field of cartilage repair is to use cocultures of different cell types, usually with articular chondrocytes, instead of choosing only one [92]. In this regard, several promising attempts have been performed with bone-marrow [93–96], synovium [97], and adipose-derived [96, 98] stem cells. IFP stem cells also present an enhanced chondrogenic potential when cocultured with articular chondrocytes [99] and most especially when cocultured in a structured manner (i.e., on top of the articular chondrocytes), instead of homogeneously mixing them, in an attempt to recapitulate the zonal characteristics of a progenitor population on top of the native articular cartilage [100].

One potential clinical concern would be the therapeutic potential of IFP stem cells obtained from a diseased OA joint. Encouragingly, studies have shown comparable chondrogenic capacities of OA-derived IFP stem cells with “healthy” cells (i.e., obtained from patients undergoing ligament reconstruction). Moreover, when cultured onto PLLA fiber membranes, OA-derived IFP stem cells generated hyaline cartilage-like grafts of approximately 2 cm diameter [32]. This property is of crucial importance for the future clinical translation of IFP stem cells, since it is necessary that even IFP cells from diseased joints are capable of enhancing cartilage repair.

Indeed, a rabbit model study showed improvement in the degree of cartilage degeneration, osteophyte formation, and subchondral sclerosis after allogeneic IFP stem cells injection 20 weeks after anterior cruciate ligament transection (ACLT) surgery to induce OA [101]. And in 2012, a therapeutic case control study level III was published using IFP cells to treat patients with OA in the knee. An average of 1.89 million cells was injected with platelet-rich plasma (PRP) as the carrier after arthroscopic procedure. PRP was further administered in a second and third round of treatment. The control group was treated with PRP injections without cells. There were significant clinical improvements evidenced by Lysholm score, Tegner activity scale, and visual analog scale (VAS) after 3 months and a last follow-up ranging from 12 to 18 months, on the same way that no major adverse events were reported. Interestingly, there was evidence that the therapy was more effective in younger patients and with early stage OA. Furthermore, although the degree of improvement was superior with cells, there was no statistical significance in the last follow-up comparing the treatment with and without cells [36].

A second study with the same group of patients, with level IV of evidence, was published one year afterwards with a final follow-up ranging from 24 to 26 months. At this point, clinical outcome was assessed with the Western Ontario and McMaster Universities Osteoarthritis Index (WOMAC), the Lysholm score, and VAS. The results were similar, although the authors noticed that improvement in WOMAC score positively correlated with the amount of injected cells [37]. It is important to highlight, though, that in this study the authors used freshly isolated cells, which did not undergo *in vitro* expansion [102].

## 6. Ontogeny Relationships between Stem Cells within the Cartilage Joint

Along with the IFP stem cells, other stem cell populations with chondrogenic capacity reside in the articular joint, such as SDSC and SFSC. Moreover, the surface of the articular cartilage itself contains a stem cell-like population that contributes to tissue appositional growth [103, 104]. These cells have been characterized in healthy articular cartilage and have been shown to possess MSC characteristics [15, 17]. Similar populations have also been identified in OA cartilage [16]. Indeed, both healthy and OA cartilage progenitor cells, which are CD105+ and CD166+, had an adipogenic and osteogenic potential similar to the bone marrow-derived MSC. Interestingly, the percentage of these cells increases with OA, comprising 4% of cells in healthy cartilage and 8% in OA cartilage [105]. Although their origin and function are still not fully elucidated, it is believed that these cartilage-derived stem/progenitor cells, expressing NOTCH-1, reside in the surface of articular cartilage, where upon injury they may migrate to the defect site in an attempt to promote tissue repair. With lesion progression, for instance in advanced stages of OA, these cells may potentially migrate throughout the cartilaginous tissue [106].

Interestingly, there is growing evidence that cartilage stem/progenitor cells are related to other stem/progenitor cells isolated from the joint. A gene expression profile study showed that cartilage progenitor cells were more closely related to synoviocytes and synovial fluid cells than to chondrocytes [107]. Moreover, *Tgfbr2*-expressing cells in the interzone have been proposed to represent a population of joint stem cells in a murine developmental model. These cells are also found in adult mice in areas such as the synovial lining of the IFP and in part of the articular cartilage [108]. Indeed, a mouse model where *Tgfbr2* is knocked-out in the limbs, fails to develop the interzone resulting in a lack of interphalangeal joints [109]. Other molecules have also been used to illustrate the ontogenetic relationship between articular cartilage and noncartilaginous tissues in the joint. For instance, articular chondrocytes and cells from the cruciate ligament and the synovium do not express *matrilin-1*; by contrast, nonarticular chondrocytes from the developing anlagen start to express *matrilin-1* when the interzone is formed [110]. Roelofs et al. recently described a stem cell population in the synovial lining and in the vascularized sublining of connective tissue derived from interzone *Gdf5*-expressing cells. Following articular cartilage injury, this population proliferates, migrates, and differentiates into chondrocytes in the cartilage-defect sites. Interestingly, cells derived from interzone *Gdf5*-expressing cells were also detected in the adult's articular cartilage, menisci, ligaments, and fat pad [57]. These data contribute to the suggestion that, as articular cartilage is ontogenetically closely related to noncartilaginous tissues in the joint, IFP stem cells could be used in future articular cartilage cell therapy trials.

**Figure 2:**
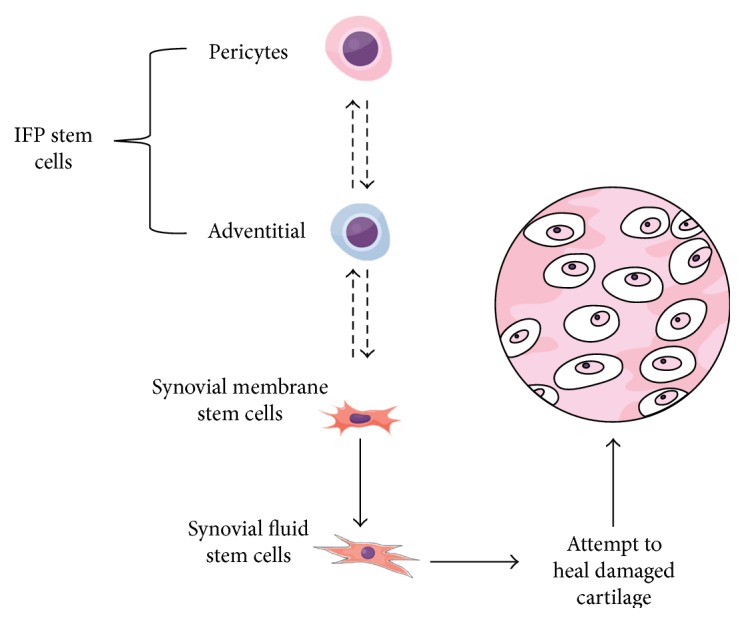
Hypothesis for a differentiation cascade between joint stem cells. The infrapatellar stem cells (IFP) would be divided into a perivascular (pericytes) and an adventitial population, with the pericytes being the most naive ones. Those would differentiate into synovial membrane stem cells. These can migrate into the synovial fluid, giving rise to synovial fluid stem cells, which would attempt to heal damaged cartilage. Dashed arrows represent more hypothetical relationships here proposed, while full arrows represent more proven ideas in the literature. This image was made using https://MindTheGraph.com.

This data also lead us to hypothesize that the IFP pericyte stem cell population described by Hindle et al. could be a tissue-resident stem cell population, as it is proposed for the subcutaneous adipose tissue [73], bone marrow [111–114], and muscle [115, 116]. Upon injury, these pericytes could be recruited, migrating to the synovium lining and into the synovial fluid in an attempt to reach and regenerate the damaged articular cartilage (Figure 2). Indeed, the IFP is highly vascularized [117], with several perivascular niches for such stem cells. Remarkably, the first observations of Maekawa et al. resemble that hypothesis, although for ACL repair [75]. Liu et al. also hypothesized that IFP stem cells participate in patella tendon repair [118]. It is important to highlight that although this hypothesis is consistent with the interzonal origin of articular cartilage and IFP, the increased number of stem cells in the synovial fluid during OA and the pattern of surface marker expression described by Hindle et al. leaves much yet to be proved. For example, by contrast, Roelofs et al. observed that synovial stem cells were not from a perivascular niche and actually gave rise to perivascular cells upon cartilage injury [57]. Furthermore, there exact relationship between IFP stem cells and SDSC must yet be elucidated.

## 7. Conclusions and Future Perspectives

It has become clear that the IFP contains progenitor cells with MSC-like characteristics, such as multilineage differentiation potential and specific surface marker expressions. Interestingly, it seems that the characteristics of these cells and their subpopulations are comparable to ADSCs, particularly in regard to the fact that IFP stem cells possess a much greater chondrogenic potential. From a translational perspective, the IFP could be easily harvested arthroscopically for cell isolation.

Cartilage cell therapy has traditionally been investigated using articular the chondrocytes, bone marrow, and subcutaneous ADSCs. The recent promising clinical trial using SDSC further encourages the therapeutic use of joint-derived stem cells, and IFP stem cells are an exciting source. These observations, along with our hypothesis on the *in vivo* role of IFP stem cells in cartilage repair, strongly support the future applications of IFP stem cells for cartilage repair clinical trials in the near future.
